# Comprehensive rehabilitation in a patient with corpus callosum syndrome after traumatic brain injury: Case report

**DOI:** 10.1097/MD.0000000000021218

**Published:** 2020-07-10

**Authors:** Xiao-li Wu, Li-xu Liu, Ling-yu Yang, Tong Zhang

**Affiliations:** Department of Neurorehabilitation, Rehabilitation Medicine of Capital Medical University, China Rehabilitation Research Centre, Beijing, China.

**Keywords:** corpus callosum, corpus callosum syndrome, diffusion tensor imaging, nerve regeneration, rehabilitation

## Abstract

**Rationale:**

Corpus callosum syndrome is a rare consequence of traumatic brain injuries. We provide a case of a patient with typical corpus callosum syndrome following a traumatic brain injury, and demonstrate neural reorganization and significant neural regeneration after comprehensive rehabilitation, using diffusion tensor imaging fiber bundle tracking.

**Patient concerns:**

We found typical clinical manifestations of damage to the corpus callosum.

**Diagnoses, Interventions, and Outcomes:**

We diagnosed a Traumatic Brain Injury (diffuse axonal injury and rupture of corpus callosum). The patient underwent a comprehensive multifaceted rehabilitation program including drug therapy, integrated physical therapy, occupational therapy, acupuncture, music therapy, computer-aided cognitive rehabilitation training, transcranial magnetic stimulation, and hyperbaric oxygen therapy. This rehabilitation program resulted in greatly improved physical and communication ability.

**Lessons:**

Comprehensive rehabilitation can significantly improve the function of patients with corpus callosum syndrome and may cause neural remodeling, as seen on diffusion tensor imaging.

## 1 Introduction

The corpus callosum connects the bilateral cerebral hemispheres, each of which has different functions. Injury to the corpus callosum results in left intentional movement apraxia, agraphia, left tactile anomic, dyslexia, alien hand syndrome, impaired left visual recognition, and left auditory hearing loss, collectively known as “corpus callosum syndrome.” This is a rare condition that occurs in patients with brain injuries and presents with a wide variety of cognitive and motor impairments. Literature is scarce regarding the response of patients with traumatic brain injuries (TBI) with corpus callosum damage to physical rehabilitation. Treadmill-based gait training (TT) has been shown to improve walking ability in a child with agenesis of the corpus callosum.^[1]^ Follow-up studies of diffusion tensor imaging (DTI) in patients with mild or severe TBI suggest that the damage to specific neural fiber bundles (right anterior radial crown, forceps major, and corpus callosum) can last over 3 months, which may be related to cognitive decline.^[2,3]^ A patient with mild TBI who had been followed up for 2 years demonstrated reorganization on DTI.^[4]^

In this case report, we describe how the patient's comprehensive rehabilitation program resulted in greatly improved physical and communication ability, and demonstrate significant neural regeneration of the corpus callosum postrehabilitation through DTI fiber bundle tracking.

## 2 Patient information

A 50-year-old male patient with a high-school diploma sustained head trauma following a car accident. He had no history of previous head trauma, hypertension, diabetes, coronary heart disease, or neurological or psychiatric disease. He also had no family history of neurological or psychiatric disease and had previously been a car maintenance worker. He smoked 10 to 20 cigarettes a day for over 20 years. He did not consume alcohol, and he was married, with 1 healthy daughter and 1 healthy son.

### 2.1 Medical history

The patient was driving out of a tunnel when he collided with a truck parked at the entrance. He immediately vomited and lost consciousness. Cranial computed tomography indicated multiple contusions and lacerations, diffuse axonal injury, and scattered cerebral hemorrhages. He was administered diuretics, mannitol immediately to reduce intracranial pressure. Several hours later, the patient underwent endotracheal intubation with simultaneous ventilation, and a naso-gastric tube and urinary catheter were inserted. Seven days after the accident, the patient was occasionally able to open his eyes and to move his fingers on his left hand slightly, but demonstrated no response to the external environment. Seventeen days after the accident, the patient was able to respond to external acousto-optic stimuli and had urinary incontinence after removal of the catheter. The patient's condition gradually improved. On the 43rd day after the accident, the patient was unable to understand instructions, could not speak or sit, and showed poor muscle strength. Rehabilitation therapy, including hyperbaric oxygen therapy, acupuncture, physiotherapy, and occupational therapy, was initiated. Approximately 2 months after the accident, the naso-gastric tube was removed, and the patient could eat independently. Four months after the accident, the patient could sit alone and had regained full muscle strength of his left limbs.

However, he sustained intellectual impairment. He could not understand or execute instructions and demonstrated weak physical strength on the right limbs. He continued physical therapy, weight-bearing standing training, limb joint training, and speech training for aphasia. However, the benefits were not obvious, and he was therefore sent to our hospital for further rehabilitation.

## 3 Clinical findings

We performed a comprehensive physical examination and found that he had severe cognitive impairment, language impairment, and disequilibrium. According to the Chinese Rehabilitation Research Center Standard Aphasia Examination (CRRCAE),^[5]^ his spontaneous language was not fluent. Listening, speaking, reading, writing, and repetition could not be completed. The LOTCA (Loewenstein Occupational Therapy Cognitive Assessment) battery score was 30, indicating a decline in comprehension and execution ability, decline in computational ability, difficulty in thinking transformation, a visuospatial disorder, and left- and right-hand agnosia. Moreover, the patient demonstrated typical left intentional movement apraxia, separate agraphia, atypical alien hand syndrome, anomia, and alexia. Although he had mild paralysis of the right limbs (Brunnstrom stage V, muscle strength grade 4^+^/5^−^), he could not remain standing, and his modified Barthel Index (MBI) score was 25. His ability to participate in life, work, studies, and societal communication was significantly reduced.

## 4 Diagnostic assessment

### 4.1 Diagnostic methods

**Figure 1:**
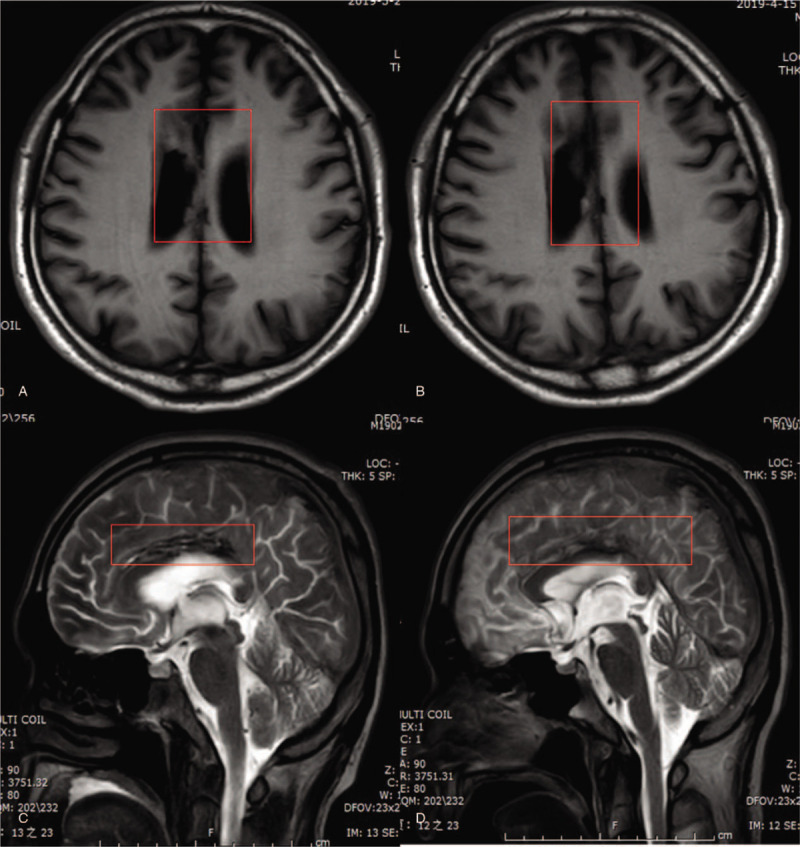
Conventional brain magnetic resonance imaging (MRI) changes. (A) and (C) show the MRI of the patient at admission. (B) and (D) show the MRI of the patient 1 month later.

Conventional brain magnetic resonance imaging (MRI) demonstrated multiple brain contusions and obvious corpus callosum rupture (Fig. 1). To clarify the effect of rehabilitation training on the patient with corpus callosum syndrome and changes in brain function, with the approval of our Institutional Review Board, and the consent of the patient's family members, we performed DTI at the time of admission and 1 month after initiating rehabilitation therapy.

DTI was performed using a 6-channel head coil on a Philips Ingenia 3.0T scanner (Philips, Best, The Netherlands). For each of the 32 noncollinear diffusion-sensitizing gradients, we acquired 90 contiguous slices parallel to the anterior commissure–posterior commissure line. The imaging parameters used were as follows (Table 1): acquisition matrix = 96 × 96; reconstructed matrix = 128 × 128 matrix; field of view (c) = 256 × 256 mm^2^; repetition time = 10,000 ms; echo time = 97 ms; sensitivity encoding factor = 2; flip angle = 90°; echo planar imaging factor = 63; b = 1000 s/mm^2^; number of excitations = 1; and slice thickness = 2 mm. Eddy current image distortions and motion artifacts were corrected with affine multiscale 2-dimensional registration, using the FMRIB Software Library (FSL, http://www.fmrib.ox.ac.uk/fsl, Oxford). The fractional anisotropy and mean diffusivity image was then calculated. Diffusion tensor reconstruction, and whole brain fiber tracking and corpus callosum fiber tracking, were conducted using Diffusion Toolkit/TrackVis.^[6,7]^ Diffusion tensor calculation was based on a linear least square-fitting algorithm.^[7]^ Tractography was performed based on a deterministic tracking algorithm ( Fiber Assignment by Continuous Tracking). An angular threshold of 45° was used to exclude high curvature streamlines.

**Table 1:**
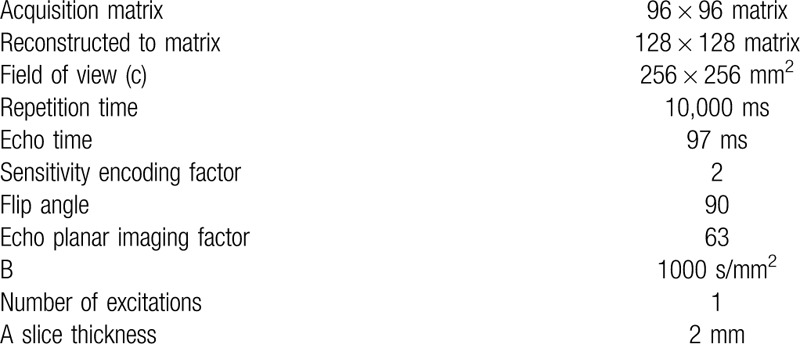
Imaging parameters.

## 5 Therapeutic intervention

### 5.1 Drug treatment (daily oral administration)

The patient was administered memantine hydrochloride, aldibenzoquinone, and oxiracetam, to improve cognition and cerebral metabolism.

### 5.2 Physical therapy (30 minutes, 5 times a week)

The patient participated in muscle strength enhancement exercises, trunk control and balance training, weight-bearing standing, joint range of motion maintenance and expansion, and body weight support treadmill training to improve movement and mobilization.

### 5.3 Occupational therapy (30 minutes, 5 times a week)

The patient participated in sitting balance improvement exercises, fine movement training, executive ability training, daily life ability training, bilateral cooperation task training, and bilateral limb coordination training to enhance bilateral hemispheric connectivity.

### 5.4 Suspension therapy (30 minutes, 5 times a week)

The patient participated in hip-knee-ankle traction training and trunk muscle traction training to improve balance and strengthen core muscles.

### 5.5 Computer-assisted cognitive impairment rehabilitation system (30 minutes, 5 times a week)

The patient participated in cognitive module training including visual agnosia, inductive classification, digital reading, picture matching memory, and number finding, to improve memory, attention, and visuospatial defects.

### 5.6 Transcranial magnetic stimulation (20 minutes, 5 times a week)

The patient received 10 minutes at the contralateral prefrontal region for cognitive improvement and 10 minutes at the contralateral temporal area for language function improvement with a stimulation frequency of 1 Hz, stimulation intensity 20% to 30%, interval time of 10 seconds, waiting time of 7.5 seconds, and sequence number of approximately 35.

### 5.7 Speech therapy (30 minutes, 5 times a week)

The patient participated in map matching, listening comprehension and after-listening retelling practice at the sentence level, writing training, induced naming, reading comprehension, and reading aloud training to improve his oral communication abilities.

### 5.8 Hyperbaric oxygen (60 minutes, 5 times a week)

The patient spent time in an air-pressurized hyperbaric oxygen chamber to improve cerebral metabolism and function.

### 5.9 Chinese acupuncture (30 minutes, 5 times a week)

The patient received “Tongdu Tiaoshen acupuncture” at Baihui (GV 20), Dazhui (GV 14), and Fengchi (GB 20), and “Xingnao Kaiqiao” acupuncture at Sishencong (EX-HN 1), Taichong (LR 3), Renzhong (DU 26), Zusanli (ST 36), Neiguan (PC 6), and Sanyinjiao (SP 6), to improve cognition.

### 5.10 Music therapy (30 minutes, 5 times a week)

The patient participated in music perception training and therapeutic singing, including imitating musical beats according to rhythm, cognitive orientation training, and lip synching. The music therapy aimed to improve his speech function through melodic singing and lip imitation, improve cognitive function through cognitive orientation training, and improve limb movements and cerebral functions by beating instruments according to a melodic rhythm.

### 5.11 Ward training

**Figure 2:**
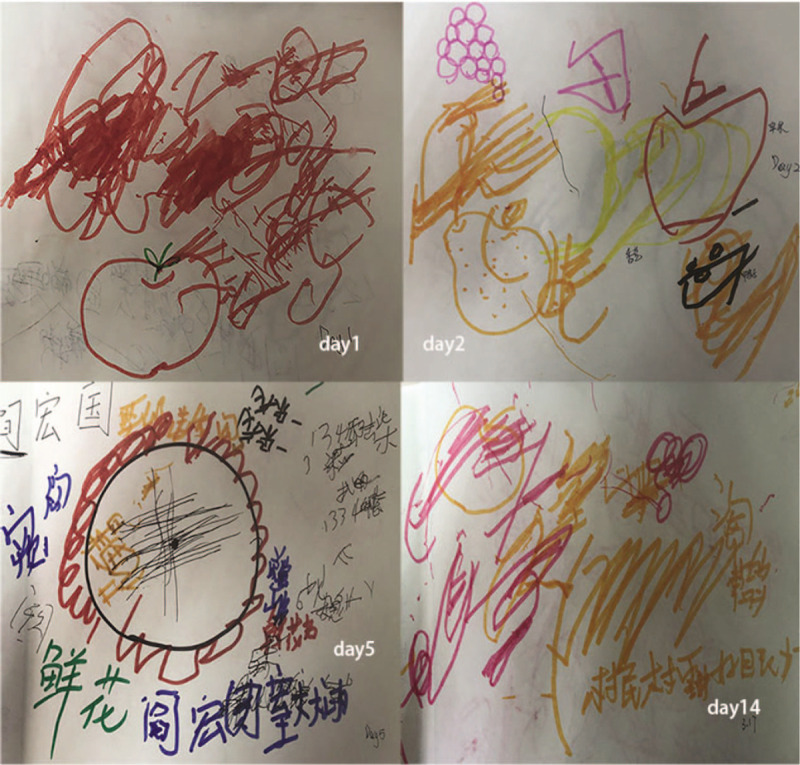
Within 2 weeks of admission, the patient practiced drawing, writing, and copying in the ward. Day 1: The patient could not imitate painting an apple, write, or copy spontaneously. Day 2: The red line indicates the apple and the black line indicates the duck. Day 5: The patient learned to draw flowers and write characters. Day 14: The patient wrote sentences spontaneously after watching television.

The patient was instructed to write, read, and copy numbers and words, perform written calculations, and draw pictures (Fig. 2).

## 6 Follow-up and outcomes

One month later, the patient could sing the Chinese songs “The East Is Red” and “My Motherland” clearly. He could answer simple questions pertinently, such as his name, age, and location, and he could name common objects. After-listening retelling ability was also partly improved. He could carry out daily activities according to instructions, such as getting a cup and drinking water, combing his hair, and washing his face with a towel. He still could not distinguish between his right and left hands. He could walk short distances with walking aids. However, his alien hand syndrome and visual agraphia did not improve (Table 2).^[8,9]^

**Table 2:**
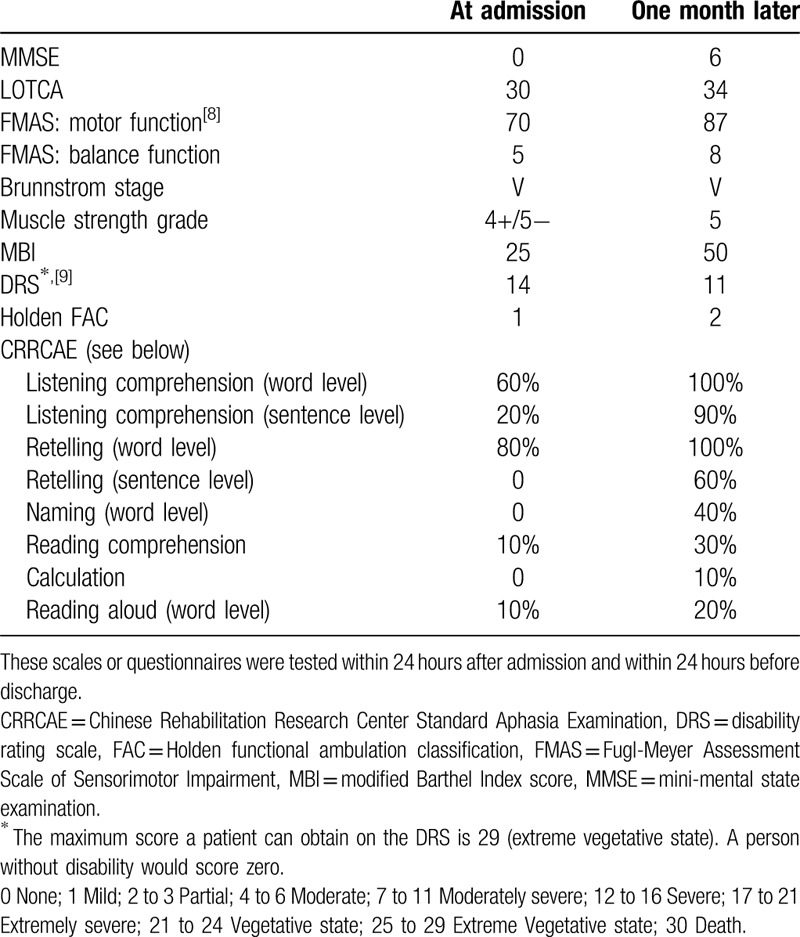
Improvement of clinical symptoms.

**Figure 3:**
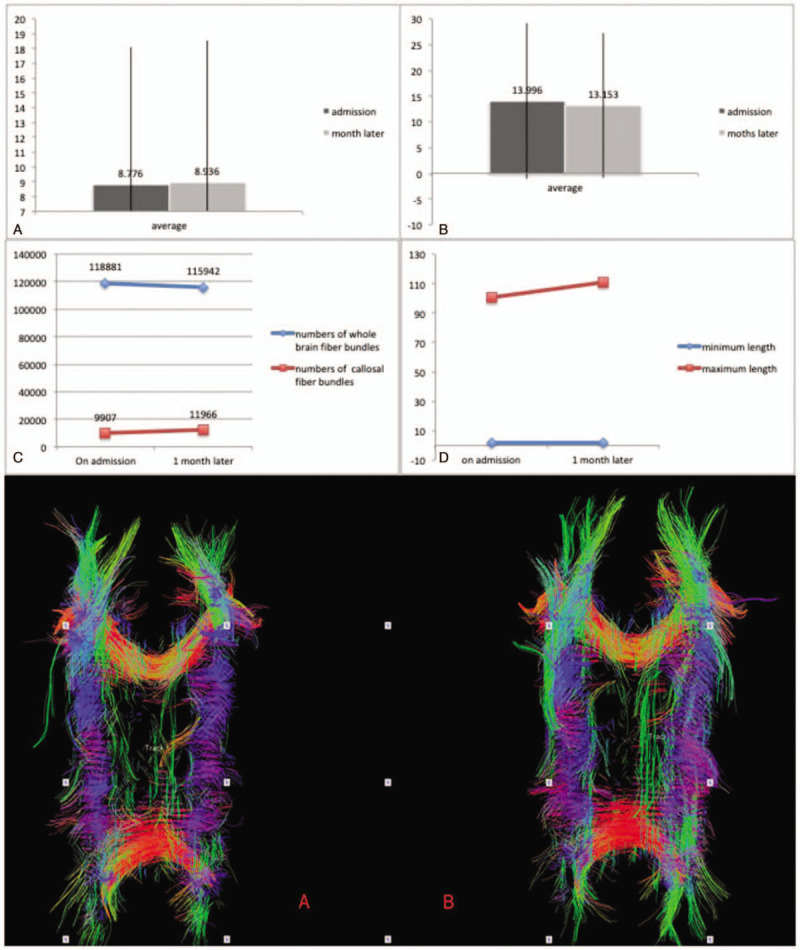
The changes in fiber bundles at the time of admission and 1 month after rehabilitation. A, The average length of the whole brain fiber bundle. Left: At admission, the average length was 8.776 ± 9.294 mm. Right: After 1 month, the average length was 8.936 ± 9.637 mm. B, The average length of the callosal fiber bundle. Left: At admission, the average length was 13.996 ± 15.08 mm. Right: After 1 month, the average length was 13.153 ± 14.041 mm. C, Blue: change in the total number of whole brain fibers. Red: change in the total number of callosal fibers. Red A: callosal fiber bundle tracking changes at admission. Red B: callosal fiber bundle tracking changes 1 month later. Red: transverse fiber bundles; green: longitudinal fiber bundles.

The DTI results of the patient at the time of admission and 1 month after initiating rehabilitation were significantly different (Fig. 3). Whole brain fiber bundle tracking indicated that the number of fiber bundles was 118,881 at admission, the minimum length was 1.4 mm, the maximum length was 100.554 mm, and the average length was 8.776 ± 9.294 mm. After 1 month, the number of fiber bundles was 115,942; the minimum length was 1.499 mm, the maximum length was 111.003 mm, and the average length was 8.936 ± 9.637 mm. The callosal fiber bundle tracking demonstrated that the total number of callosal fibers was 9907, the minimum length was 1.499 mm, the maximum length was 100.554 mm, and the average length was 13.996 ± 15.08 mm. One month later, the total number of visible fibers increased to 11,966; the minimum length was still 1.499 mm, the maximum length increased to 111.003 mm, and the average length was 13.153 ± 14.041 mm. Although the number of whole brain fiber bundles decreased, the number and length of the callosal fiber bundle increased after rehabilitation, especially the fibers connecting the bilateral hemispheres. As shown in Fig. 3, the red transverse fiber bundles connecting the bilateral hemispheres and the green longitudinal fibers increased.

## 7 Discussion

The patient demonstrated typical signs of corpus callosum rupture posttraumatic brain injury, including alien hand syndrome, impaired left visual recognition, segregated writing, and left intentional movement apraxia, according to the literature.^[10]^ We used DTI to determine whether rehabilitation affected neurodegeneration and brain remodeling. After 1 month of rehabilitation, we not only found that the clinical manifestations of the patient were improved, but also that neural regeneration could be seen on DTI. The possible mechanism behind this is that the corpus callosum is an arcuate fiber, which breaks and demyelinates easily after injury, without degeneration or necrosis of neurons. If diagnosis and treatment are timely, myelin will regenerate, and symptoms will improve.

Many studies have shown reduced fractional anisotropy in the corpus callosum of patients with diffuse axonal injury on DTI, even when conventional MRI findings are normal.^[11–15]^ Some studies also suggest that these microscopic changes in the corpus callosum are related to severity and outcome of the injuries.^[3]^ In our patient, MRI and DTI showed severe corpus callosum rupture. The corpus callosum is believed to connect the bilateral cerebral hemispheres, as some studies have shown that cortical excitability in the unaffected motor cortex could increase after transient suppression of cortical excitability caused by transection of the corpus callosum.^[16]^ Furthermore, Neumann et al^[17]^ showed that training of the impaired forelimb after TBI could enhance neuroplasticity at functionally remote regions, including the hippocampus in Emx1 null mice lacking a corpus callosum, indicating that this may have implications for promoting overall recovery of function after TBI. Clinically, a case report of cerebral concussion after TBI from a South Korean study, in which the cingulate gyrus was damaged, was identified by DTI. Two years later, DTI follow-up revealed neurological reorganization between the injured cingula and the brainstem cholinergic nucleus.^[4]^ These suggested that the reorganization of the corpus callosum may be possible after traumatic brain injury.

Research on neural rehabilitation is in a bottleneck period, which has been effective in promoting novel research on conditions including TBI, stroke, and autism; however, the mechanism of neural plasticity remains to be further elucidated. A single means of rehabilitation cannot provide the maximum rehabilitation effect, so comprehensive rehabilitation is more commonly used in current clinical practice.^[18,19]^ In our patient, in addition to drug therapy, we integrated physical therapy, occupational therapy, acupuncture, music therapy, computer-aided cognitive rehabilitation training, transcranial magnetic stimulation, and hyperbaric oxygen to improve limb function, speech, cognition, and capability in daily life. Physical therapy, occupational therapy, cognitive training, and hyperbaric oxygen have proven effective in the rehabilitation of brain injuries. Additionally, transcranial magnetic stimulation and music therapy, as new rehabilitation therapies, have been widely recognized in cognitive and speech rehabilitation research.^[20–23]^ Therefore, patients with corpus callosum injuries may benefit from comprehensive rehabilitation therapy. However, this paper is limited to 1 case report, and it would be better if there were more rehabilitation studies on corpus callosum injury and follow-up using DTI. In addition, there may be differences in the rehabilitation methods and techniques among different hospitals. Before admission to our hospital, the patient underwent limited rehabilitation for cognitive and language in the early stage, apart from physical therapy. Therefore, once the TBI patient's condition is stable, early comprehensive rehabilitation intervention may be more effective.

In conclusion, reorganization of the corpus callosum was seen in our patient with a corpus callosum rupture following TBI after appropriate rehabilitation therapy. A comprehensive, multifaceted rehabilitation program can result in good patient outcomes. This result recommends the use of a multifaceted rehabilitation program and the use of DTI to monitor recovery in TBI patients with corpus callosum syndrome.

## Acknowledgments

The authors thank Yunlei Wang for English-language editing. They thank the patient and his family for their support of our study.

## Author contributions

**Funding acquisition:** Tong Zhang.

**Supervision:** Tong Zhang.
